# Association of neighborhood-level disadvantage beyond individual sociodemographic factors in patients with or at risk of knee osteoarthritis

**DOI:** 10.1186/s12891-024-08007-7

**Published:** 2024-11-07

**Authors:** Jessica Peoples, Jared J. Tanner, Emily J. Bartley, Lisa H. Domenico, Cesar E. Gonzalez, Josue S. Cardoso, Catalina Lopez-Quintero, Elizabeth A. Reynolds Losin, Roland Staud, Burel R. Goodin, Roger B. Fillingim, Ellen L. Terry

**Affiliations:** 1 Biobehavioral Nursing Science, University of Florida, Gainesville, FL USA; 2 Department of Clinical and Health Psychology, Gainesville, FL USA; 3 Department of Psychology, University of Alabama at Birmingham, Birmingham, AL USA; 4 Department of Biobehavioral Health, Penn State University, University Park, PA USA; 5 Department of Epidemiology, University of Florida, Gainesville, FL USA; 6 Department of Medicine, University of Florida, Gainesville, FL USA; 7 Department of Anesthesiology, Washington University in St. Louis, St. Louis, MO USA; 8 Pain Research and Intervention Center of Excellence (PRICE), College of Nursing, University of Florida, 1225 Center Drive, PO Box 100197, Gainesville, FL 32610-0197 USA; 9 Community Dentistry and Behavioral Science, University of Florida, Gainesville, FL, USA

**Keywords:** Area deprivation index, Race, Knee osteoarthritis, Socioeconomic factors

## Abstract

**Objective:**

Lower socioeconomic status (SES) is a risk factor for poorer pain-related outcomes. Further, the neighborhood environments of disadvantaged communities can create a milieu of increased stress and deprivation that adversely affects pain-related and other health outcomes. Socioenvironmental variables such as the Area Deprivation Index, which ranks neighborhoods based on socioeconomic factors could be used to capture environmental aspects associated with poor pain outcomes. However, it is unclear whether the ADI could be used as a risk assessment tool in addition to individual-level SES.

**Methods:**

The current study investigated whether neighborhood-level disadvantage impacts knee pain-related outcomes above sociodemographic measures. Participants were 188 community-dwelling adults who self-identified as non-Hispanic Black or non-Hispanic White and reported knee pain. Area Deprivation Index (ADI; measure of neighborhood-level disadvantage) state deciles were derived for each participant. Participants reported educational attainment and annual household income as measures of SES, and completed several measures of pain and function: Short-form McGill Pain Questionnaire, Western Ontario and McMaster Universities Osteoarthritis Index, and Graded Chronic Pain Scale were completed, and movement-evoked pain was assessed following the Short Physical Performance Battery. Hierarchical linear regression analyses were used to assess whether environmental and sociodemographic measures (i.e., ADI 80/20 [80% least disadvantaged and 20% most disadvantaged]; education/income, race) were associated with pain-related clinical outcomes.

**Results:**

Living in the most deprived neighborhood was associated with poorer clinical knee pain-related outcomes compared to living in less deprived neighborhoods (*p*s < 0.05). Study site, age, BMI, education, and income explained 11.3–28.5% of the variance across all of the individual pain-related outcomes. However, the ADI accounted for 2.5–4.2% additional variance across multiple pain-related outcomes.

**Conclusion:**

The ADI accounted for a significant amount of variance in pain-related outcomes beyond the control variables including education and income. Further, the effect of ADI was similar to or higher than the effect of age and BMI. While the effect of neighborhood environment was modest, a neighborhood-level socioenvironmental variable like ADI might be used by clinicians and researchers to improve the characterization of patients’ risk profile for chronic pain outcomes.

## Introduction

Knee osteoarthritis (OA) is among the top reasons older adults seek medical care due to chronic pain and functional decline, which contributes to multiple years lived with disability [1–3]. In the United States, prevalence estimates indicate approximately 14 million adults have symptomatic knee osteoarthritis [4] and the prevalence rates are expected to increase as the population ages [5]. In 2017, annual knee osteoarthritis treatment costs were estimated up to $15 billion [6]. Further, Bedenbaugh and colleagues [6] found that the total annual healthcare costs for individual patients with knee osteoarthritis were $7,707 *more than* the annual healthcare costs of matched controls. The burden of symptomatic knee OA is not shared equally; it disproportionately affects at-risk groups including racially and ethnically minoritized persons [7, 8] and economically disadvantaged individuals [8, 9]. Black patients have lower inpatient admission rates and lower rates of knee OA surgery compared to White patients, but Black patients have a statistically higher rate of healthcare expenditures ($25,974) compared to White patients’ expenditures ($22,913) [10].

The individual or combined measures of educational attainment, household income, or occupation are often used as a proxy for individual-level social and economic advantage or disadvantage (socioeconomic status; SES) [11, 12]. There is evidence of an association between chronic pain and SES, such that chronic pain is inversely related to SES [13–15]. However, some pain studies failed to find an association [16, 17], in part, perhaps because varying methods are used to define and measure SES [18–21]. Another limitation is that, as Khalatbari & Blyth [22] reported, few studies investigating socioeconomic inequality and pain have been grounded in a particular theoretical approach. While Craig [23] has called for the development of theoretical models focused on the social factors of pain, the preponderance of theoretical models focus predominantly on the interpersonal (i.e., relating to relationships) and social communication model of pain [24, 25]. Blyth and colleagues [26] argue that based on a theory-driven approach (e.g., the biopsychosocial model), there is a need for more research incorporating socioenvironmental factors, which are the least investigated component of the biopsychosocial model.

Socioenvironmental factors typically include both the social and economic components of the lived environment [27]; they can be operationalized using census tract data, postal codes, or other geographic area measures to determine neighborhood-level built environments [28]. Within the United States, census data include economic indicators such as employment status, housing-quality, and poverty; these data can be used to examine the impact of living in disadvantaged neighborhoods independent of individual-level socioeconomic factors [29, 30]. The Area Deprivation Index (ADI) is a validated approach to measure neighborhood-level disadvantage, using U.S. census data [29, 31, 32]. The ADI is a publicly-available mapping tool that ranks neighborhoods based on their socioeconomic conditions [32]. One significant advantage to using the ADI is that it can be utilized to analyze complex geospatial data without the need for geospatial technical skills. Thus, the ADI could easily be integrated into healthcare systems and research protocols to provide additional information about potential pain outcomes. However, research is needed to assess whether the ADI is associated with pain outcomes above and beyond measures of individual-level SES. One advantage of using the ADI, rather than obtaining information about patient’s individual income and education, is that disclosure of individual-level socioeconomic information may be subject to response bias due to social desirability and lack of knowledge about family finances, as well as difficulty in comparing educational quality across a patient’s educational attainment. For example, even within one geographical area, the quality of the education may not be comparable with an adjacent geographic area even within the same postal code [33, 34].

Further, neighborhood-level disadvantage may be concentrated in geographic areas where racial residential segregation may disadvantage marginalized families by limiting housing options to areas that are under-resourced and where infrastructure disinvestment persists [35]. The neighborhood environments of marginalized racially segregated communities (e.g., non-Hispanic Black persons) have been reported to be more disadvantaged than White neighborhoods. Their disadvantages are associated with poor health and pain-related outcomes due in part to limited access to resources and opportunities [36–41].

Prior research investigating neighborhood disadvantage frequently dichotomized neighborhoods into least deprived areas relative to those in the most deprived areas (e.g., top 15–20%) in order to investigate its influence on health and pain outcomes [29, 42–48]. Cross-sectional studies consistently find that living in the most deprived neighborhoods is associated with adverse health and pain outcomes including: worse knee pain and function prior to and 6 months after total knee replacement [44], greater pain interference and mental health symptoms [7, 49], increased health care utilization [29, 46], increased risk for chronic pain [37], poorer health outcomes [45, 50], multimorbidity [30], and reduced life expectancy [51, 52] compared to those in the least deprived neighborhoods. In short, living in the most disadvantaged neighborhoods has a particularly striking effect on or association with health outcomes [7]. There has, however, been limited investigation on the influence of neighborhood disadvantage on participants from marginalized racial and ethnic groups [18].

Therefore, we investigated whether living in neighborhoods with greater deprivation is associated with poorer knee OA-related pain outcomes, above self-reported individual-level measures of SES (e.g., education, income). In addition, we examined the interaction between neighborhood environment and race to determine if neighborhood factors differentially influenced pain-related outcomes in non-Hispanic Black vs. White individuals. The current hypotheses are: Hypothesis 1: adults living in the most deprived neighborhoods would have worse pain-related outcomes compared to adults living in the least deprived neighborhoods, controlling for study site. Hypothesis 2: neighborhood environment would account for variance in clinical pain-related outcomes above self-reported individual-level measures of SES (i.e., education, income), accounting for study site, age, and body mass index (BMI). Hypothesis 3: race would moderate the relationship between neighborhood disadvantage and pain-related outcomes.

## Patients and methods

### Study overview

The current study is a cross-sectional sub-study of a larger longitudinal observational cohort study entitled *Understanding Pain and Limitations in OsteoArthritic Disease – Second Cycle*,* (UPLOAD-2)*. The UPLOAD-2 study was designed to investigate the mechanisms underlying racial and ethnic group differences in knee pain in non-Hispanic Black adults and non-Hispanic White adults. Data for the UPLOAD-2 study were collected at two sites (i.e., University of Florida (UF) and The University of Alabama at Birmingham (UAB)) between August 2015 and May 2017. The study was approved by the institutional review boards at the University of Florida and The University of Alabama at Birmingham.

### Participants

The current sample comprised 188 community dwelling adults who self-identified as non-Hispanic Black or non-Hispanic White and self-reported symptoms of knee OA.

### Procedures

Participants completed a standardized telephone screening to confirm initial eligibility. Self-reported sociodemographic (e.g., race/ethnicity, sex, age) and health and pain history data (e.g., symptoms of knee OA) were collected during the screening to determine initial eligibility. Participants who met the inclusion criteria were invited to the clinic. During the clinic visit, participants were informed about the study procedures and signed an informed consent, prior to any data collection. Participants then completed additional sociodemographic (e.g., income, education), health and pain questionnaires. Body mass index (BMI) was calculated from in-clinic measurements of height and weight. Participants self-reported symptoms of knee OA. Questionnaires assessing clinical pain and disability were administered electronically. Prior to the end of the first visit, participants completed the Short Physical Performance Battery (SPPB) and movement-evoked pain was assessed. There are significant socioeconomic and other demographic differences between the non-Hispanic Black and non-Hispanic White participants in the UPLOAD-2 data [53].

### Measures

*Area Deprivation Index (ADI).* The ADI is a validated measure of neighborhood-level disadvantage [29, 31, 32]. The ADI incorporates 17 social and economic variables (e.g., housing-quality, poverty, income, education) drawn from the U.S. census data (2015 American Community Survey) [29] to generate a decile value that represents neighborhood-level economic indicators for every census tract using postal codes and ranked into relative quintiles based on statewide distributions [29]. A decile value of one indicates the least amount of disadvantage, while a value of ten indicates maximum neighborhood-level disadvantage. In the U.S., census tracks are used for compiling national population data. Census tracts generally have a population size between 1,200 and 8,000 people, with an optimum size of 4,000 people.

Previous research demonstrated that people who live in disadvantaged areas have worse pain, poorer physical function, and a greater burden of health risk factors and comorbidities when compared to people who live outside those areas [7, 54]. Following previous work [42, 43, 47], we created a dichotomous variable for the ADI with the least (80%) and most (20%) disadvantaged ADI comprising separate categories. The most disadvantaged were coded as 1 and the least disadvantaged were coded were coded as 2.

*Education.* Self-reported level of education attained was assessed using the following 6 categories: less than High School, High School Degree, Associate’s Degree, Bachelor’s degree, Master’s Degree, and Doctoral/Professional.

*Income*. Annual household income was assessed using 10 levels of income starting at $0–$9,999; $10,000–$19,999; $20,000–$29,999; $30,000–$39,999; $40,000–$49,999; $50,000–$59,999; $60,000–$79,999; $80,000–$99,999; $100,000–$149,999; and the last category was $150,000 or higher. The income levels were categorized in this format to better capture higher proportions of participants in the lower income levels compared to lower proportions of participants in the higher income levels. Mean z-scores from the sample of education level and income level were used to create the combined education/income variable [55] to better represent socioeconomic status than either variable alone.

### Outcome variables

*Short-form McGill Pain Questionnaire-revised (SF-MPQ-2).* SF-MPQ-2 is a 22 item questionnaire that measures the quality and intensity of pain [56]. A modified version of the SF-MPQ-2 was administered, instructing participants to rate the intensity of their pain experience in their most bothersome knee, during the past week. An 11-point numeric rating scale (0 = “none” to 10 = “worst possible”) was used to compute an average across all questions.

*Western Ontario and McMaster Universities Osteoarthritis Index (WOMAC)*. The WOMAC is a 24-item questionnaire that assesses knee OA pain, stiffness, and physical function symptoms over the past 48 h [57]. The WOMAC pain (range 0–20) and physical function (range 0–68) subscales were included in data analysis. Severity of symptoms are rated on a five-point Likert scale (0 indicating “None” and 4 indicating “Extreme”) and higher scores on these two subscales reflect greater pain and poorer physical functioning, respectively. The WOMAC is validated and reliable (Cronbach’s α: pain subscale = 0.89 and physical function subscale = 0.97) [57].

*Graded Chronic Pain Scale (GCPS)*. The GCPS is a seven-item questionnaire that assesses knee pain intensity and interference (i.e., disability) over the previous 6 months [58]. Items were averaged and multiplied by 10 to obtain index scores for pain intensity and disability. Higher scores reflect greater symptomatology [58]. Cronbach’s α for the GCPS was 0.91.

*Short Physical Performance Battery (SPPB).* The SPPB is a standardized measure of lower-extremity function including tests of: standing balance, 4-meter gait speed, and chair-rising [59]. Each measure is scored from 0 indicating worst performance to 4 indicating best performance. Lower scores indicate poorer function [59, 60]. The total SPPB score was used in analyses.

*Movement-evoked Pain (MEP)*. Immediately after each SPPB test, participants rated their overall knee pain on a scale from 0 indicating no knee pain to 100 indicating the most intense knee pain imaginable, as a measurement of movement-evoked pain [61].

### Data analysis

Data were analyzed using SPSS 28.0 (IBM, Chicago, IL). All data were checked for normality by visual inspection of distributions and using the Shapiro-Wilk test [62]. None of the variables followed a normal distribution based on the Shapiro-Wilk test (all *p* values < 0.02). Data were also assessed for distributional form using visual inspection of the Q-Q plots. Outliers were identified in the following variables: SF-MPQ Total, SPPB Total Score, and Movement-Evoked Pain (MEP). Next, we ran the skewness value and divided by standard error of skewness; only 3 variables (SF-MPQ Total, SPPB Total Score, and MEP) failed the skewness test of normality and were greater than the critical value of ± 1.96 [63]. There were no other patterns that would otherwise indicate violation of assumptions. As a *post hoc* analysis, SF-MPQ Total, SPPB Total Score, and MEP scores were Blom transformed with regression analyses repeated for these three measures. The results and interpretation did not change for any of the variables, thus untransformed scores and original analyses were used. Parametric statistics were therefore used to analyze the data, and no participants were excluded. All testing was two sided using a 0.05 level of significance. Given our theory-grounded, targeted hypotheses, we did not correct for multiple comparisons [64, 65]. Differences in sociodemographic and clinical characteristics between ADI groups were assessed using chi-square (χ^2^) and independent samples t-tests. Differences across ADI and outcome variables were assessed using a one-way between-groups analysis of covariance (ANCOVA).

**Fig. 1:**
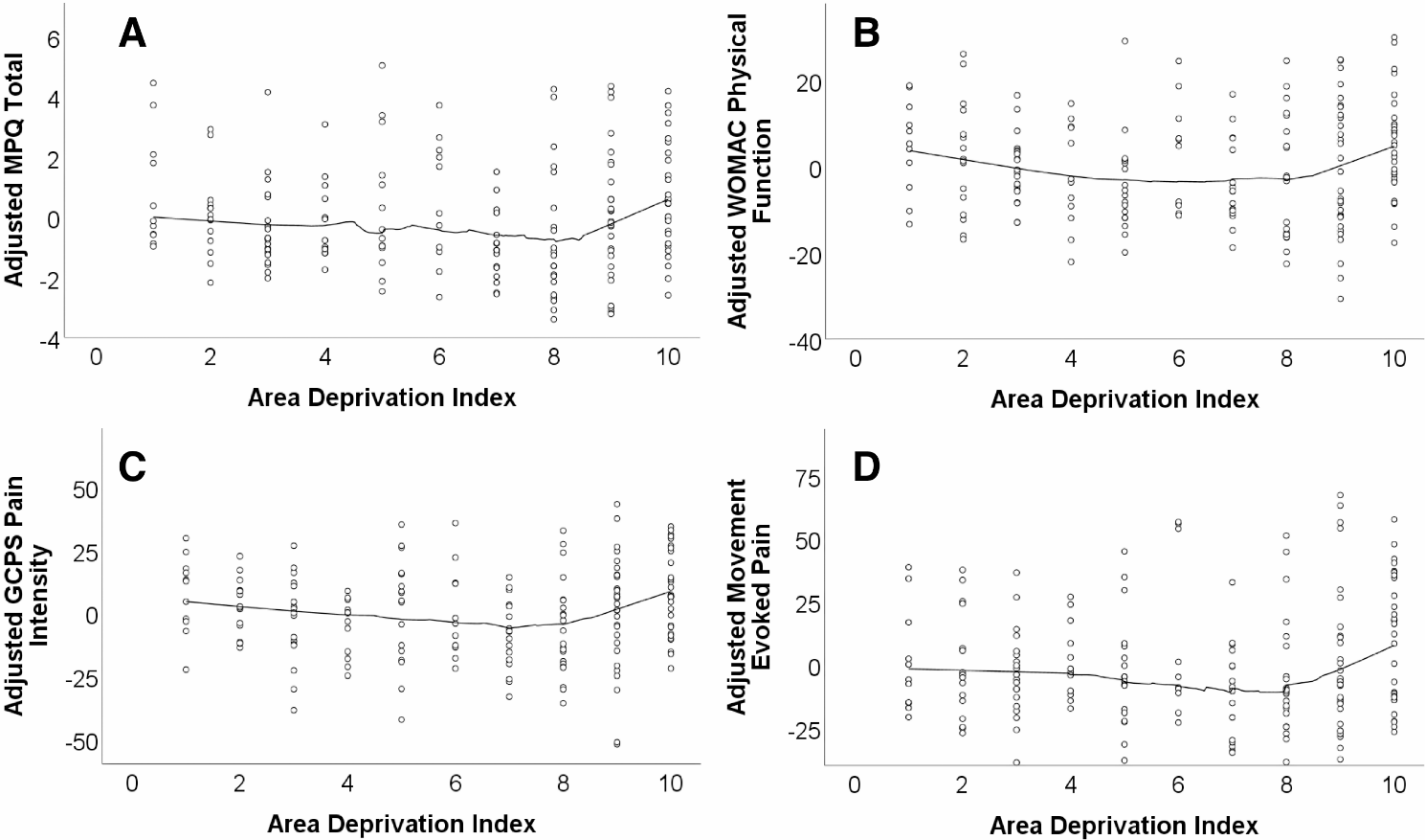
Scatterplots showing the relationship between area deprivation index and pain-related outcomes. *Note*: MPQ Total = Short-form McGill Pain Questionnaire - revised; WOMAC = Western Ontario and McMaster Universities Osteoarthritis Index; GCPS = Graded Chronic Pain Scale; Covariates = study site, age, BMI, education/income, and race

Scatterplots were used to show the relationship between ADI and pain-related outcomes, adjusted for study site, age, BMI, and education/income. Plots include the line fit using locally estimated scatterplot smoothing (LOESS) (Fig. 1).

Hierarchical linear regression analyses were used to assess whether socioenvironmental and sociodemographic measures (i.e., ADI, race) were associated with pain-related clinical outcomes (i.e., clinical pain, pain-related disability, physical function, and movement-evoked pain), after controlling for the influence of study site, age, body mass index (BMI), and education/income. Covariates were selected based on their influence on pain [66]. Analyses were carried out in four steps. First, study site, age, BMI, and education/income were entered in the regression model as control variables (Block 1). Secondly, ADI was added to the regression model (Block 2). Thirdly, race was added to the regression model (Block 3). Finally, the race by ADI interaction was added to the model (Block 4) to assess if race moderated the ADI grouping. As a *post hoc* analysis, we ran the hierarchical regressions with education and income separately in the model rather than the combined z-score.

The pain variables failed to fit the assumption of normality (Shapiro-Wilk *p* values < 0.02). However, given the sample size, we used parametric statistical approaches for the main analyses as described in the methods.

## Results

### Participant characteristics

Participant characteristics are presented for the overall sample and by ADI 80/20 (Table 1). There were 188 participants with an average age of 58 (range: 45–78 years old) with 63% (*n* = 119) self-identifying as female. A little over half of the participants self-identified as non-Hispanic Black 51.6% (*n* = 97). 41.5% (*n* = 78) of the participants reported a high school diploma, 36% (*n* = 67) of the sample were married, and 29.3% (*n* = 54) self-reported an annual income of less than $10,000. Eleven participants were missing SF-MPQ-2, one participant was missing WOMAC pain, and one participant was missing movement-evoked pain. Participants with missing data were excluded from analyses. Participants who lived in areas with greater neighborhood deprivation were more likely to be non-Hispanic Black (*p* < .01), have lower income (*p* < .01), have lower educational attainment (*p* < .01), and less likely to be married (*p* < .01) compared to participants who lived in less deprived neighborhoods. In addition, participants from the University of Florida study site were more likely to have higher ADI (*p* < .01).

**Table 1:** Demographics and clinical characteristic of participants across ADI group

	Overall *N* = 188	ADI 20 *N* = 61	ADI 80 *N* = 127	
	M or N (SD or %)	M or N (SD or %)	M or N (SD or %)	*p*
**Age** (years)	58.0(7.8)	56.9(7.0)	58.5(8.1)	0.18
Study Site*				< 0.01
UF	121(64.4)	52(85.2)	69(54.3)	
UAB	67(35.6)	9(14.8)	58(45.7)	
**Sex**				0.84
Female	119(63.3)	38(62.3)	81(63.8)	
Male	69(36.7)	23(37.7)	46(36.2)	
Race*				< 0.01
Non-Hispanic Black	97(51.6)	41(67.2)	56(44.1)	
Non-Hispanic White	91(48.4)	20(32.8)	71(55.9)	
Income*				< 0.01
$0-$9,999	54(29.3)	28(46.7)	26(21.0)	
$10,000 - $19,999	25(13.6)	10(16.7)	15(12.1)	
$20,000 - $29,999	26(14.1)	7(11.7)	19(15.3)	
$30,000 - $39,999	8(4.3)	3(5.0)	5(4.0)	
$40,000 - $49,999	13(7.1)	2(3.3)	11(8.9)	
$50,000 - $59,999	16(8.7)	1(1.7)	15(12.1)	
$60,000 - $79,999	15(8.2)	6(10.0)	9(7.3)	
$80,000 - $99,999	10(5.4)	2(3.3)	8(6.5)	
$100,000 - $149,999	12(6.5)	0(0.0)	12(9.7)	
≥150,000	5(2.7)	1(1.7)	4(3.2)	
**Education**				0.05
Some School, < high school	13(6.9)	7(11.5)	6(4.7)	
High School Degree	78(41.5)	31(50.8)	47(37.0)	
Associates Degree	33(17.6)	10(16.4)	23(18.1)	
Bachelor’s Degree	37(19.7)	7(11.5)	30(23.6)	
Master’s Degree	20(10.6)	6(9.8)	14(11.0)	
Doctoral/Professional	7(3.7)	0(0.0)	7(5.5)	
**Employment**				0.68
Employed	73(38.8)	21(34.4)	52(40.9)	
Temporary leave	4(2.1)	1(1.6)	3(2.4)	
Not Employed	26(13.8)	10(16.4)	16(12.6)	
Retired	45(23.9)	12(19.7)	33(26.0)	
Disabled	29(15.4)	13(21.3)	16(12.6)	
Other	2(1.1)	1(1.6)	1(0.8)	
Marital status*				0.01
Married	67(36.0)	13(21.7)	54(42.9)	
Widowed	14(7.5)	4(6.7)	10(7.9)	
Divorced	53(28.5)	16(26.7)	37(29.4)	
Separated	9(4.8)	6(10.0)	3(2.4)	
Never married	37(19.9)	18(30.0)	19(15.1)	
Living with partner	6(3.2)	3(5.0)	3(2.4)	
**Insurance status**				0.07
No	30(16.0)	15(24.6)	15(11.8)	
Yes	157(83.5)	46(75.4)	111(87.4)	
**Knee pain duration**				0.56
<6 months	10(5.3)	3(4.9)	7(5.6)	
6 to 12 months	15(8.0)	5(8.2)	10(7.9)	
1 to 3 years	47(25.1)	20(32.8)	27(21.4)	
3 to 5 years	27(14.4)	8(13.1)	19(15.1)	
>5 years	88(47.1)	25(41.0)	63(50.0)	
**BMI** (kg/m^2^)	32.0(7.7)	32.0(8.6)	32.1(7.2)	0.93

Note: *p < .05. M = Mean; N = Number; UF = University of Florida; UAB = University of Birmingham at Alabama; BMI = body mass index

### Scatterplot

As shown in Fig. 1A-D, the scatter plots were overlaid with the line fit using LOESS; these figures showed an upward trend with a positive relationship between ADI and pain-related outcomes at the upper 20th percentile with the relationships appearing to be non-linear. Therefore, as in prior work [42, 43, 47], we included the ADI 80% least disadvantaged versus 20% most disadvantaged variable in all subsequent analyses.

### ADI differences in pain, disability and functional performance

When only adjusting for study site, there were significant ADI differences in all pain outcome measures (*ps* < 0.05; Table 2). Participants who lived in areas with greater neighborhood deprivation had poorer pain-related outcomes, and poorer functional performance compared to participants who lived in less deprived neighborhoods.

**Table 2:** Inferential statistics for measures of clinical pain, pain-related disability and function

	ADI 20 *N* = 61	ADI 80 *N* = 127	Comparison	
	M (SD)	M (SD)	F	η_*p*_^2^
SF-MPQ-2 Total	3.4(2.2)	2.3(2.2)	15.22**	0.08
WOMAC Pain	9.0(4.6)	7.3(4.1)	10.31**	0.05
WOMAC Physical Function	29.8(15.1)	22.8(13.8)	15.71**	0.08
GCPS Pain Intensity	65.6(23.2)	51.0(21.6)	25.03**	0.12
GCPS Interference	57.8(29.9)	42.4(29.3)	13.66**	0.07
SPPB Total Score (0–12)	8.8(1.7)	9.6(1.7)	5.93*	0.03
Movement-Evoked Pain	28.8(28.6)	19.4(23.7)	13.60**	0.07

Note: **p < .01, *p < .05. ADI = area deprivation index; SF-MPQ- 2 = Short-form McGill Pain Questionnaire - revised; WOMAC = Western Ontario and McMaster Universities Osteoarthritis Index; GCPS = Graded Chronic Pain Scale; SPPB = Short Physical Performance Battery; CI = confidence interval; Covariates = study site. SF-MPQ-2 *n* = 177; WOMAC pain *n* = 187; Movement-Evoked Pain *n* = 187

### Hierarchical regression

Refer to Table 3 for all regression models for pain-related outcome variables. Study site, age, BMI, education, and income accounted for 11.3–28.5% of the variance across all of the individual pain-related outcomes. In significant models, the ADI accounted for 2.5–4.2% of the variance above and beyond that explained by the combined study site, age, BMI, education, and income. In Block 2, the statistically significant standardized beta coefficients for ADI (-0.173 to − 0.225) were lower than the standardized beta coefficients for education and income (-0.121 to − 0.311), other than for movement-evoked pain (-0.121). The ADI was generally similar to or higher than the standardized beta coefficients for age (-0.085 to − 0.230) and BMI (0.053 to − 0.303). The ADI*race interaction in Block 4 was not statistically significant in any model. However, Race in Block 3 was statistically significant in most models, except for SPPB Total. Below are summaries of the models for Blocks 2 and 3.

**Table 3:** Predictors of clinical pain, pain-related disability, physical function, and movement-evoked pain from education/income, area deprivation index 80/20, and race: hierarchical linear regression analyses

	Model 1: SF-MPQ-2 Total	Model 2: WOMAC Pain	Model 3: WOMAC Physical Function	Model 4: GCPS Pain Intensity	Model 5: GCPS Interference	Model 6: SPPB Total Score	Model 7: Movement-Evoked Pain
Independent Variables							
Block 1: Control Variables							
Study Site	0.087	0.067	0.086	0.060	− 0.006	0.185*	0.209**
Age	− 0.159*	− 0.205**	− 0.135	− 0.249**	− 0.211**	− 0.118	− 0.113
BMI	0.142*	0.123	0.204**	0.108	0.124	− 0.301**	0.049
Education/Income	− 0.357**	− 0.299**	− 0.304**	− 0.370**	− 0.264**	0.319**	− 0.178*
*R*^*2*^	0.233	0.206	0.213	0.285	0.175	0.221	0.113
*F*	13.072**	11.812**	12.406**	18.192**	9.694**	13.008**	5.794**
Block 2: Social/Environmental							
Study Site	0.144*	0.105	0.144*	0.129*	0.048	0.154*	0.276**
Age	− 0.148*	− 0.194**	− 0.119	− 0.230**	− 0.196**	− 0.127	− 0.085
BMI	0.148*	0.127	0.208**	0.113	0.128	− 0.303**	0.053
Education/Income	− 0.307**	− 0.267**	− 0.254**	− 0.311**	− 0.218**	0.292**	− 0.121
ADI 80/20	− 0.177*	− 0.124	− 0.187**	− 0.225**	− 0.173*	0.101	− 0.214*
*R*^*2*^	0.258	0.219	0.242	0.326	0.200	0.230	0.150
*ΔR*^*2*^	0.025	0.013	0.029	0.042	0.025	0.008	0.037
*F*	11.920**	10.139**	11.635**	17.631**	9.078**	10.858**	6.406**
*ΔF*	5.840*	2.944	9.204**	11.292**	5.632*	1.979	7.968*
Block 3: Race							
Study Site	0.112	0.085	0.116	0.083	0.048	0.166*	0.247**
Age	− 0.121	− 0.177*	− 0.097	− 0.195**	− 0.196**	− 0.136	− 0.074
BMI	0.136*	0.123	0.201**	0.102	0.128	− 0.300**	0.047
Education/Income	− 0.244**	− 0.230**	− 0.205**	− 0.232**	− 0.218**	0.272**	− 0.075
ADI 80/20	− 0.133	− 0.097	− 0.150*	− 0.165**	− 0.173*	0.085	− 0.176*
Race Group	− 0.261**	− 0.154*	− 0.208**	− 0.334**	− 0.182*	0.088	− 0.201**
*R*^*2*^	0.316	0.239	0.279	0.421	0.228	0.236	0.184
*ΔR*^*2*^	0.058	0.020	0.037	0.095	0.028	0.007	0.034
*F*	13.116**	9.417**	11.667**	21.928**	8.898**	9.337**	6.786**
*ΔF*	14.418**	4.576*	9.204**	29.574**	6.602*	1.562	7.527**
Block 4: Interaction							
Race group*ADI 80/20	0.034	0.057	0.036	0.029	0.003	− 0.039	0.086
*R*^*2*^	0.318	0.242	0.280	0.422	0.228	0.238	0.191
*ΔR*^*2*^	0.001	0.003	0.001	0.001	0.000	0.001	0.007
*F*	11.232**	8.161**	10.004**	18.753**	7.585**	8.022**	6.050**
*ΔF*	0.268	0.715	0.297	0.249	0.002	0.336	1.517

Note: **p ≤ .01; *p ≤ .05. The reported are standardized beta weights. ADI = area deprivation index; SF-MPQ- 2 = Short-form McGill Pain Questionnaire - revised; WOMAC = Western Ontario and McMaster Universities Osteoarthritis Index; GCPS = Graded Chronic Pain Scale; SPPB = Short Physical Performance Battery; Covariates = study site, age, BMI, education/income

#### SF-MPQ-2

In the partially adjusted model (Block 2), there was evidence that ADI group was negatively associated with SF-MPQ-2, explaining 2.5% of the variance (t=-2.42, *p* = .017); indicating more deprivation was associated with greater pain. In the Block 3 model, white race (t=-3.80, *p* < .001), lower BMI (t = 2.05, *p* = .042), and higher education/income (t=-3.48, *p* < .001) were associated with lower pain, but ADI group (t=-1.86, *p* = .065) and study site (t = 1.63, *p* = .106) were not significant.

#### WOMAC Pain

The ADI group variable was not significantly associated with WOMAC Pain (t=-1.72, *p* = .088). In the Block 3 model, white race (t=-2.18, *p* = .031), higher age (t=-2.54, *p* = .012), and higher education/income (t=-3.22, *p* = .002) were associated with lower pain. ADI group variable (t=-1.33, *p* = .184) and study site (t = 1.21, *p* = .229) were not significant in Block 3.

#### WOMAC physical function

The ADI group variable was significantly associated with WOMAC physical function, explaining 2.9% of the variance (t=-2.64, *p* = .009), suggesting more neighborhood deprivation was associated with greater functional impairment. In the Block 3 model, less deprived ADI group (t=-2.12, *p* = .036), white race (t=-3.03, *p* = .003), lower BMI (t = 3.06, *p* = .003), higher education/income (t=-2.96, *p* = .004) were associated with lower pain. Study site (t = 1.70, *p* = .092) and age (t=-1.44, *p* = .153) were not significant predictors.

#### GCPS Pain Intensity

The ADI group variable was significantly associated with GCPS pain intensity in the partially adjusted model (t=-3.36, *p <* .001; 4.2% of variance explained). Therefore, higher neighborhood deprivation was associated with greater pain intensity. In the Block 3 model, less deprived ADI group (t=-2.60, *p* = .010), white race (t=-5.44, *p <* .001), higher age (t=-3.22, *p* = .002), higher education/income (t=-3.74, *p* < .001) were associated with lower pain. Study site (t = 1.36, *p* = .176) and BMI (t = 1.74, *p* = .084) were not significant.

#### GCPS interference

The ADI group was negatively associated with GCPS interference, explaining 2.5% of the variance (t=-2.37, *p =* .019), indicating higher neighborhood deprivation was associated with greater interference. In the Block 3 model, white race (t=-2.57, *p =* .011), higher age (t=-2.53, *p =* .012), and higher education/income (t=-2.44, *p =* .016) were associated with lower pain. ADI group (t=-1.925, *p =* .056), study site (t = 0.322, *p =* .748), and BMI (t = 1.80, *p =* .074) were not significant.

#### Short performance functional battery (SPPB)

The ADI group was not significantly associated with SPPB (t = 1.41, *p* = .161). In the Block 3 model, ADI group (t = 1.17, *p* = .245) and race group (t = 1.25, *p* = .213) were not significant. However, University of Florida (UF) study site (t = 2.37, *p* = .019), lower BMI (t=-4.44, *p* < .001), and higher education/income (t = 3.80, *p* < .001) were associated with higher functional performance on the SPPB. Age was not significant (t=-1.96, *p* = .052).

#### Movement-Evoked Pain

The ADI was negatively associated with movement-evoked pain (t=-2.82, *p* = .005; 3.7% of variance explained), suggesting that living in a more deprived neighborhood is associated with greater pain during movement. In the Block 3 model, less deprived ADI group (t=-2.32, *p* = .021), white race (t=-2.74, *p* = .007), and University of Florida (UF) study site (t = 3.39, *p* < .001) were associated with lower pain. Age (t=-1.02, *p* = .307), BMI (t = 0.67, *p =* .503), and education/income (t=-1.01, *p* = .314) were not significant.

*Post hoc* analyses including the other control variables (study site, age, BMI) with education and income not combined, indicated similar patterns for all models.

## Discussion

Evidence demonstrates an association between chronic pain and socioeconomic status [13, 15, 20, 67–73]. However, there is a need for research to investigate social environmental factors (e.g., neighborhoods) to better understand community burden of chronic pain risk and pain outcomes [17, 74, 75]. The Area Deprivation Index (ADI) is a socioenvironmental variable that ranks neighborhoods based on socioeconomic factors. This could be a useful tool to capture neighborhood socioeconomic factors associated with knee OA-related outcomes.

The current study investigated: (1) whether living in neighborhoods with greater deprivation was associated with greater knee OA-related pain, disability, and function, controlling for study site; (2) whether the impairment in knee OA-related outcomes is associated with ADI above self-reported individual-level measures of SES (i.e., education, income), accounting for study site, age, and BMI; and (3) whether the interaction between neighborhood environment and race account for differences in knee OA-related outcomes. The first hypothesis indicated that participants living in the neighborhoods with the most deprivation would have higher levels of clinical pain and pain-related disability, and demonstrated lower levels of physical function compared to participants living in the least deprived neighborhoods. Our first hypothesis was fully supported; we found that compared to the least deprived neighborhoods, participants living in the most disadvantaged neighborhoods generally reported experiencing higher levels of clinical pain and poorer pain-related physical function on pain outcomes. These findings are consistent with prior research, which demonstrate relationships between neighborhood disadvantage and poorer pain-related outcomes [9, 14]. The neighborhood environments of disadvantaged communities have characteristics such as mobility and transportation barriers, limited access to community resources, higher rates of environmental pollution, reduced access to healthy food options, prescription medications, and health care creating a milieu of increased stress and deprivation that adversely affects health and chronic pain outcomes [37, 38, 76–81]. Therefore, living in communities with high social disadvantage compounds the deleterious effects of chronic pain and health outcomes due to the persistent exposure of greater adversity, fewer resources, and stressful psychosocial and economic circumstances [82, 83]. Evidence shows a significant association between neighborhood deprivation and clinical pain, even after accounting for household SES [84, 85].

Our data are consistent with existing literature demonstrating that individual-level self-reported SES [e.g., educational attainment, income] is a risk factor that contributes to chronic pain-related outcomes [13, 15, 19, 20, 67, 69]. Specifically, several cross-sectional studies have reported lower SES is associated with OA prevalence, greater pain severity, and higher rates of pain-related disability in persons with OA [86–88]. In addition, longitudinal data with 5 and 6 years of follow-up identified the trajectories and risk profiles of pain in individuals with or at risk of knee OA and found that lower educational attainment (i.e., high school graduate or less vs. college graduate) was among the factors associated with trajectories characterized by greater pain [89–91]. While we do not dispute that income and education are important independent indicators of a patient’s risk for knee pain outcomes [92], knee osteoarthritis is a condition that affects older adults who are more likely to be retired. In these instances, some SES metrics (e.g., previous occupation) may not reflect a patient’s current financial position [16, 17, 93]. Further, studies of individual-level SES typically include one variable [92]. In contrast, the ADI is an index of 17 measures of neighborhood relevant variables [29, 32], making it a more robust measure of social disadvantage.

Our second hypothesis was partially supported. We found that ADI accounted for an additional 2.5–4.2% variance in clinical pain and pain-related disability (SF-MPQ-2, WOMAC Physical function, GCPS, and MEP), but was not significantly associated with WOMAC pain or SPPB physical function. Once we added in race group in Block 3, race group was significant for all outcome measures, except SPPB. Accounting for race, living in the top 20% (most deprived) neighborhoods was significantly associated with lower WOMAC Physical Function, higher GCPS pain and interference, and greater reports of movement-evoked pain. The ADI accounted for a modest, but statistically significant amount of variance in pain-related outcomes beyond sociodemographic factors, including education and income. Further, the effect of ADI was similar to, or higher than, the effect of age and BMI for multiple pain outcomes.

Given the cumulative effect of social neighborhood disadvantage, we were surprised that living in a disadvantaged area was not associated with functional performance, above individual-levels of SES. Specifically, we expected that since participants from disadvantaged neighborhoods reported more severe symptomatic knee pain and greater interference during daily activities, this group would demonstrate poorer functional performance compared to their peers from more affluent neighborhoods. Mehta and colleagues [94] found that patients with advanced knee osteoarthritis showed impaired functional performance on the SPPB. However, while we found no differences in the functional performance tasks, participants living in disadvantaged neighborhoods rated their pain as more severe when engaged in functional performance activities, suggesting that they may push themselves to complete activities despite the pain.

In respect to our findings, this relationship was modest. Indeed, although the variance accounted for by the ADI did not reach clinical significance (typically defined as approximately 2-point reduction or 30% decrease in a measure [95–97], these findings are still considered meaningful. Jordan and colleagues [17] conducted a prospective study in 5993 people with follow-up at 3 years and found that living in a deprived area accounted for 2% of the variance in prevalence and incidence of disabling pain. Blyth [74] contends that, despite the small variations observed in Jordan et al.’s [17] study, research focused on lived environments has the potential to impact change at the community level to reach a larger number of people, as opposed to a smaller population at the individual level.

Our data provide evidence that neighborhood-level disadvantage is associated with clinical OA-related pain outcomes, above self-reported individual level socioeconomic factors. While both individual SES and neighborhood deprivation are associated with worse pain and interference [9], neighborhood-level characteristics are an important factor to consider when examining OA-related pain, disability, and physical function outcomes. Specifically, living in the lower resourced neighborhoods was associated with worst knee OA-related pain and disability outcomes. Other researchers who used the ADI to predict health outcomes have shown similar deleterious associations in neurodegeneration and cognitive decline (e.g., cortical thinning) in persons who live in the most disadvantaged (i.e., top quintile) neighborhoods compared to persons who live in the least disadvantaged neighborhoods [42, 43, 47].

The third hypothesis tested whether race would moderate the relationship between neighborhood disadvantage and pain-related outcomes. This hypothesis was not supported. Race was significantly related to OA-related outcomes, indicating Black participants reported greater pain, but this relationship was not significant for SPPB function. However, race did not moderate the relationship between neighborhood environment and OA-related pain outcomes. Therefore, there was insufficient evidence that the association of neighborhood environment with pain-related outcomes differed by race. These findings parallel previous research. For example, in two studies Green and colleagues showed lower neighborhood SES was associated with poorer chronic pain outcomes to the same extent in both Black and White older adults seeking pain care [39, 40]. Overall, results from the current study align with prior work and suggest neighborhood-level environment, particularly in the 20% most disadvantaged areas, is associated with clinical pain and pain-related disability across race groups and beyond individual-level self-reported SES.

These findings provide evidence that neighborhood-level disadvantage represents a risk factor for poor OA-related outcomes. Further, neighborhood environment appears to be a social component of marginalized communities that likely provides helpful information about the risk profile for patients’ pain outcomes. In fact, increased neighborhood disadvantage has been shown to be associated with increased adverse health events and all-cause mortality in many countries [45, 50, 52]. However, neighborhood disadvantage metrics are underused in both clinical and research settings. Neighborhood-level maps such as the Neighborhood Atlas [32], a free, and readily accessible neighborhood metric, can be utilized to obtain a patient’s area deprivation index (ADI) using their address, allowing providers to evaluate patients’ risk for chronic OA-related pain outcomes. There are other metrics that can also be used (e.g., the Social Vulnerability Index) [53, 98]. Neighborhood disadvantage or vulnerability-informed care could be used to help identify at-risk patients and enable effective allocation of attention, resources, and monitoring to support the unique needs of patients living in the most disadvantaged areas.

A patient’s address is standard demographic information obtained when patients establish clinical care and is more likely to be known compared to household income. Some patients might be hesitant to disclose income or educational attainment [99] and, in those instances, the ADI might serve as a proxy. Further, when SES data are reported, the combination of individual-level SES and ADI could enable health care systems to use a holistic approach to evaluate patients’ risk for greater pain-related outcomes.

Study strengths include a racially diverse sample of non-Hispanic Black and non-Hispanic White participants, in whom we collected multiple well-validated clinical pain-related measures. There were also notable limitations to this study. First, the study was not designed to test the current aims and therefore, may not have been adequately powered to test the interaction between race and ADI. Second, although other studies have used the 80/20 split, separating the data into two groups leads to loss of information. However, the practice of comparing least deprived areas to most deprived areas is common practice in this area of research and aligns with prior studies [29, 42–47]. Third, in considering the use of ADI in clinical settings, patients with unstable housing situations living in the most disadvantaged areas may move frequently, making it difficult to maintain accurate and updated records on patients’ neighborhood environments. In addition, the ADI does not capture individuals’ histories, including how long they lived in the area nor where they lived in the past. However, Knighton [100] found that patients in highly deprived neighborhoods often move to areas with similarly disadvantaged neighborhoods and therefore, a current address within a 1-3-year timeframe is useful in patients seeking clinical care.

Future research could focus on investigating metrics of individual SES and sociodemographic data combined with environmental data (e.g., neighborhood resources, geospatial measures) that can be easily integrated into clinical settings and research protocols to identify patients’ pain risk profiles. This could allow more comprehensive understanding of pain disparities among minoritized groups. Further, future studies should consider the inclusion of other minoritized groups (e.g., Hispanic groups) in order to generalize conclusions drawn regarding the association between neighborhood disadvantage and knee OA-related outcomes in chronic pain.

In summary, the current study revealed that participants living in the 20% most disadvantaged neighborhoods reported experiencing higher levels of clinical pain and poorer pain-related physical function. Further, neighborhood-level disadvantage modestly impacts pain-related outcomes (e.g., clinical pain and disability) above and beyond individual-level self-reported SES measures. While race and individual SES remain salient factors in patients’ risk for chronic pain and poor health outcomes, considering socioenvironmental resources can be valuable in understanding the complex set of interactions and social determinants of health that comprise a patient’s risk profile for poorer OA-related outcomes. The clinical application of specific metrics of socioenvironmental resources as an assessment tool to help identify patients living in highly disadvantaged areas who could benefit from additional proactive interventions for chronic pain in future research.

## Data Availability

The data that support the findings of this study are available from the corresponding author [ELT], upon reasonable request.
